# *SYN2* is an autism predisposing gene: loss-of-function mutations alter synaptic vesicle cycling and axon outgrowth

**DOI:** 10.1093/hmg/ddt401

**Published:** 2013-08-15

**Authors:** Anna Corradi, Manuela Fadda, Amélie Piton, Lysanne Patry, Antonella Marte, Pia Rossi, Maxime Cadieux-Dion, Julie Gauthier, Line Lapointe, Laurent Mottron, Flavia Valtorta, Guy A. Rouleau, Anna Fassio, Fabio Benfenati, Patrick Cossette

**Affiliations:** 1 Department of Experimental Medicine, University of Genova, Viale Benedetto XV 3, Genova 16132, Italy; 2 Department of Neuroscience and Brain Technologies, Fondazione Istituto Italiano di Tecnologia, Via Morego 30, Genova 16163, Italy; 3 Centre of Excellence in Neuromics and CHUM Research Center, Université de Montréal, CHUM-Hôpital Notre-Dame, 1560 Sherbrooke Est, Montréal, QC, CanadaH2L 4M1; 4 S. Raffaele Sci. Institute and Vita-Salute University, Via Olgettina 58, Milano 20132, Italy

## Abstract

An increasing number of genes predisposing to autism spectrum disorders (ASDs) has been identified, many of which are implicated in synaptic function. This ‘synaptic autism pathway’ notably includes disruption of *SYN1* that is associated with epilepsy, autism and abnormal behavior in both human and mice models. Synapsins constitute a multigene family of neuron-specific phosphoproteins (*SYN1-3*) present in the majority of synapses where they are implicated in the regulation of neurotransmitter release and synaptogenesis. Synapsins I and II, the major Syn isoforms in the adult brain, display partially overlapping functions and defects in both isoforms are associated with epilepsy and autistic-like behavior in mice. In this study, we show that nonsense (A94fs199X) and missense (Y236S and G464R) mutations in *SYN2* are associated with ASD in humans. The phenotype is apparent in males. Female carriers of *SYN2* mutations are unaffected, suggesting that *SYN2* is another example of autosomal sex-limited expression in ASD. When expressed in *SYN2*  knockout neurons, wild-type human Syn II fully rescues the *SYN2* knockout phenotype, whereas the nonsense mutant is not expressed and the missense mutants are virtually unable to modify the *SYN2* knockout phenotype. These results identify for the first time *SYN2*  as a novel predisposing gene for ASD and strengthen the hypothesis that a disturbance of synaptic homeostasis underlies ASD.

## INTRODUCTION

Autism spectrum disorders (ASDs) are a heterogeneous group of disorders characterized by impaired social relationships, rigid and repetitive behavior, restricted interests and abnormal language development (1). Genetic factors are playing an important role in ASD (2–4), and an increasing number of genes predisposing to the disease have been identified over the past 10 years. Notably, the majority of the ASD-predisposing genes thus far identified encode for synaptic proteins, such as the postsynaptic proteins neuroligins 3 and 4 (*NLGN3*, *NLGN4*), their cytoplasmic interactors *SHANK2* and *SHANK3* and their presynaptic partner neurexin-1 (*NRXN1*) (5–13). Genetic variants in another member of the neurexin superfamily, contactin-associated protein-like 2 (*CNTNAP2*) have also been associated with ASD (14–17), as well as mutations in two additional synaptic genes, namely *IL1RAPL1* and *RIMS3/NIM3* (18,19). The fact that the majority of the identified ASD-predisposing genes encode for synaptic proteins led to the ‘synaptic autism pathway’ hypothesis, holding that ASD is due to abnormal synaptic function and neural connectivity in the time window in which neuronal circuits are remodeled by experience (20,21).

Epileptic seizures are observed in up to one-third of ASD individuals (1,22) and autistic features are commonly observed in severe forms of epilepsy (23). Recently, mutations in human *SYN1* have been reported to represent a common basis for both ASD and epilepsy (24,25). Although most of the known epilepsy predisposing genes implicate voltage-gated or ligand-gated ion channels (26), defects in synaptic proteins implicated in neurotransmitter release and synaptic vesicle (SV) trafficking have been frequently associated with an epileptic phenotype in mouse models (26–30).

Synapsins (Syns) are a family of neuron-specific SV phosphoproteins implicated in synaptic transmission and plasticity (31). In mammals, Syns are encoded by three distinct genes (*SYN1*, *SYN2* and *SYN3)* located on chromosomes Xp11.23, 3p25.2 and 22q12.3, respectively. Alternative splicing generates distinct isoforms, termed *a*, *b* and *b-like* composed of a mosaic of individual and shared domains. Synapsins I and II are selectively expressed at nerve terminals in mature neurons, whereas the expression of Syn III is downregulated in mature neurons and the protein is not strictly confined to synaptic terminals. Synapsins contribute to the clustering of SVs and regulate their trafficking between the recycling pool (RP) and the readily releasable pool (RRP), thus defining SV availability for release in a phosphorylation-dependent fashion. Perturbation of Syn function in a variety of models leads to disruption of the organization of SV pools in the presynaptic compartment and to an increase in synaptic depression, underlining the role of Syns in sustaining neurotransmitter release in response to high-frequency activity (31–33). Moreover, recent studies have shown that Syns also play a role in the post-docking stages of release and their perturbation leads to an imbalance between the activities of excitatory and inhibitory neurons (34–37). Besides the well-documented role at mature synapses, a plethora of data also implicates Syns in neuronal development, from the early stages of neurite sprouting to the regulation of synapse formation and refinement (38).

Each *SYN* gene has been inactivated in mice (27,28,35,39–41). Despite the absence of gross defects in brain anatomy, *SYN1* and *SYN2* knocked out (KO) mice exhibit spontaneous seizures, whereas *SYN3* KO mice are not epileptic. Interestingly, both *SYN2* and *SYN3* KO mice also display cognitive impairments, suggesting that Syns could be involved in the regulation of higher brain functions (42–45). Consistent with these observations, we recently showed that deletion of *SYN1*, *SYN2* or *SYN3* widely impairs social behaviors, resulting in an ASD-related phenotype that is more pronounced in *SYN2* KO mice. Interestingly, social impairments in both *SYN1* and *SYN2* KO mice were observed before the onset of seizures, suggesting that the behavioral impairments are not merely a consequence of the epileptic phenotype (46).

Based on these observations, we hypothesized that mutations in *SYN2*, similarly to mutations in its functionally related gene *SYN1*, can predispose to ASD or epilepsy. *SYN2* was found among the five neural genes whose single nucleotide polymorphisms (SNPs) contribute to epilepsy predisposition in a screening of 279 prime candidate genes in 2717 cases of epilepsy (47). An intronic polymorphism in *SYN2* was also found to be associated with idiopathic epilepsy (48).

In this study, we report three mutations in *SYN2* (one nonsense and two missense) identified by direct sequencing of the entire gene that are associated with ASD. While the nonsense variant was not expressed in either cell lines or neurons, the translated proteins of the missense variants were normally targeted to nerve terminals, although they were unable to rescue the *SYN2* KO phenotype in terms of SV pool dynamics and/or axon elongation. Taken together, these results support the view that rare genetic variations in *SYN2* predispose to ASD and emphasize the key role of the Syn family of presynaptic proteins in the pathogenesis of the disease.

## RESULTS

### Identification of nonsense and missense *SYN2* mutations in ASD patients

**Figure 1.**
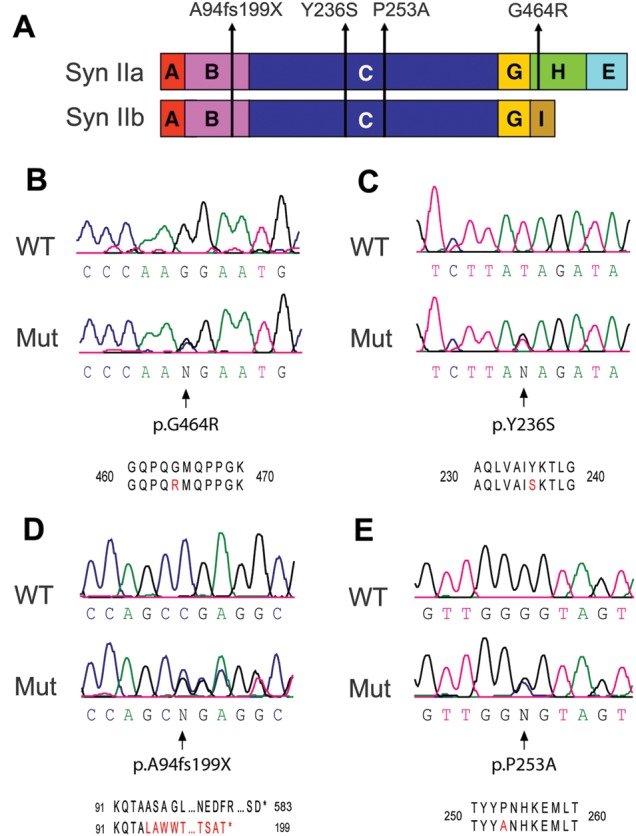
Nonsense and missense mutations identified in *SYN2* and associated with ASD. (**A**) Modular structure of the *a* and *b* splicing isoforms of Syn II and location of the four mutations linked to ASD/epilepsy identified in this study. While the p.A94fs199X mutation [domain (B)] and the p.Y236S/p.P253A mutations [domain (C)] involve NH_2_-terminal domains shared by the two isoforms, the p.G464R mutation is localized to the proline-rich H domain that is specific for Syn IIa. (**B**–**E**) Sequence analysis showing the identification of the p.G464R (B), p.Y236S (C), p.A94fs199X (D) and p.P253A (E) mutations in *SYN2*.

We sequenced the coding and splicing junction regions of *SYN2* in 190 cases with ASD and 143 cases with partial epilepsy. We found a total of four novel genetic variations, including one nonsense mutation in one ASD case (p.A94fs199X), two missense mutations in ASD cases (p.Y236S and p.G464R) and one missense mutation in an epilepsy case (p.P253A) (Fig. 1A–E). These genetic variants were not reported in the public databases (1000 Genomes, *dbSNPs*). The three missense mutations were located in exons 6 and 13 of *SYN2*. Sequencing of these exons in 90 additional ASD cases did not reveal any variations. Sequencing the entire *SYN2* coding regions in 335 control individuals did not show any rare variant, suggesting that the novel genetic variants found in ASD and epilepsy are indeed mutations or at least very rare variants. Statistical analysis showed that the excess of rare missense variants is significant in the ASD, but not in the epilepsy cohort (*P* = 0.0471 and *P* = 0.299, respectively). All *SYN2* mutation carriers with ASD were male and the mutation was transmitted by the unaffected mother (Tables 1 and 2). We are not aware of any affected sibling with ASD in *SYN2* mutation carriers. Considering the positive association of *SYN2* mutations with ASD, we further analyzed the impact of mutant *SYN2 in vitro*.

**Table 1.** SYN2 missense mutations identified in ASD and epilepsy

Ind	Sex	Exon	cDNA	Protein	Phenotype	Inheritance
26 755	H	6	c.757C>G	p.P253A	Epilepsy	Mother
24 053	H	2	c.282>del7pb	p.A94fs199X	ASD	Mother
24 579	H	6	c.707A>C	p.Y236S	ASD	Mother
17 205	H	13	c.1390G>C	p.G464R	ASD	Mother

**Table 2.** ASD phenotypes of *SYN2* mutation carriers

Subject ID	Sex	Ethnicity	Mutation	Diagnosis at time of recruitment	ASQ^a^	ADOS-G^b^	ADI-R^c^
24 579	Male	French-Canadian	p.Y236S	ASD, higher functioning	16	C:4/S:7/T:11	N/A
24 053	Male	French-Canadian	p.A94fs199X	Autism	33	C:4/S:10	C:14/S:28
17 205	Male	Asian	p.G464R	Autism	N/A	C:6/S:14	C:14/S:26/St:5/D:4

^a^ASQ, Autism Screening Questionnaire: ASQ cutoff score >15 = autism.

^b^ADOS-G, Autism Diagnostic Observation Schedule-Generic: C, communication (autism cutoff = 4; autism spectrum = 2); S, social interaction (autism cutoff = 7; autism spectrum = 4); T, total (autism cutoff = 12; autism spectrum = 7).

^c^ADI-R, Autism Diagnostic Interview-Revised: C, communication; S, social; St, stereotype; D, development.

### *In silico* predictions of the impact of *SYN2* mutations

Synapsins are composed of a mosaic of shared, evolutionarily conserved, domains (A, B, C, E) and of individual domains (D, F, G, H, J) (31). The nonsense mutation p.A94fs199X is caused by a deletion (del 282_288fs) that leads to an early frameshift, followed by a premature stop codon (pos.598). If translation occurred, the frameshift would cause the expression of a protein truncated at position 199, and aberrant in the sequence from amino acid 95 to amino acid 198, thus removing the majority of the structure of both the *a* and *b* isoforms of *SYN2* (Fig. 1A and D; Supplementary Material, Fig. S1A). The missense mutation Y236S involves the highly conserved central C-domain of Syn II and the G464R mutation hits the proline-rich H-domain that is specific for the Syn IIa isoform (Fig. 1A). Thus, while the A94fs199X *SYN2* mutation is likely to have a dramatic impact on the translation and/or functional properties of the gene product, the effects of the missense mutations (Y236S and G464R) were hardly predictable by *in silico* analysis. Indeed, while the Y236 residue can show a conservative substitution with F in mammals, the G464 residue is conserved between man, rat and mouse, but not in lower vertebrates (Supplementary Material, Fig. S1B). Analysis of the missense substitutions by POLYPHEN-2 software (http://genetics.bwh.harvard.edu/pph/) predicted a significant impact on the human Syn II ortholog (hSyn II) protein function for the G464R mutation (HumDiv, 0.97 score; HumVar, 0.78 score), but not for the Y236S mutation.

### Expression pattern of hSyn II mutants in HeLa cells and primary neurons

**Figure 2.**
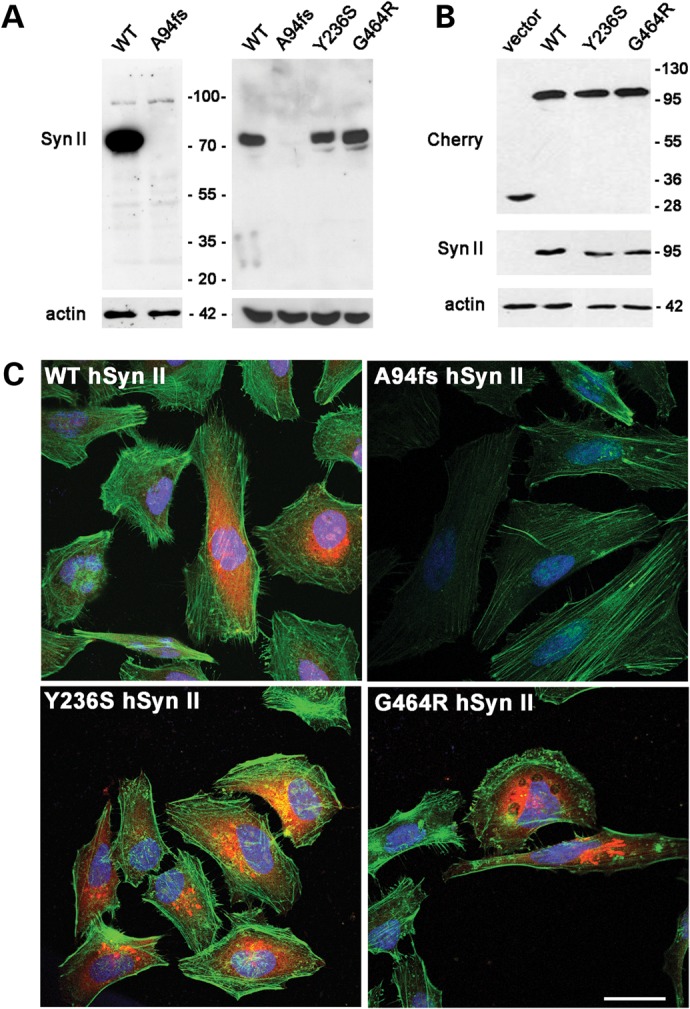
The nonsense hSyn II mutant is not expressed, while the missense mutants are expressed at normal levels in both HeLa cells and *SYN2* KO neurons. (**A**) Expression of the A94fs199X-hSyn II mutant in HeLa cells (*left*) and hippocampal neurons (*right*). Hela cells were transfected with either WT-hSyn II or A94fs199X-hSyn II in a pCAGGS-ires-Tomato vector and harvested 48 h later. *SYN2* KO hippocampal neurons were nucleofected at 0 DIV with pCAGSS-ires-Tomato vector encoding for either WT-hSyn II, A94fs199X-hSyn II, Y236S-hSyn II or G464R-hSyn II and lysed at 3 DIV. The expression of the A94fs199X-hSyn II isoform was undetectable in both Hela cells and neurons. (**B**) Expression of mCherry-tagged missense hSyn II mutants in HeLa cells. mCherry-tagged variants of either WT-hSyn II, Y236S-hSyn II or G464R-hSyn II were transiently transfected in HeLa cells, with the empty vector (−) used as control. Cells were harvested 48 h after transfection. The expression of recombinant hSyn II variants was analyzed by SDS page and immunoblotting (A and B) using a monoclonal antibody (Mab19.21) directed to the hSyn IIa/IIb sequence S^12^SFIAN located NH_2_ terminal to the frameshift (72). In B, the expression of the mCherry fusion proteins was double checked using anti-Cherry antibodies. Mutant hSyn II isoforms were expressed at levels similar to those of the WT protein in both cell systems. In all immunoblots, actin staining was used as a control of equal loading. (**C**) Confocal images of Hela cells transfected with mCherry-tagged variants of WT-hSyn II, A94fs199X-hSyn II, Y236S-hSyn II or G464R-hSyn II and counterstained with AlexaFluor-488 phalloidin to visualize the F-actin cytoskeleton. WT-hSyn II and missense variants were expressed in the cytoplasm of transfected cells, whereas the expression of A94fs199X-hSyn II was totally absent. Scale bar, 30 μm.

We first investigated the expression of the nonsense and the Y236S/G464R missense mutations in a human cell line (HeLa cells), as well as in murine hippocampal neurons prepared from *SYN2* KO mice (Fig. 2). While transfection of HeLa cells or nucleofection of primary hippocampal neurons with the wild-type (WT) hSyn II promoted a reliable expression of the protein at the expected molecular mass, the expression of the A94fs199X-hSyn II mutant was totally undetectable in either cell system and no immunoreactive band in the expected molecular mass range of 20 kDa was seen, even under conditions of gel overloading (Fig. 2A, left). In contrast, the expression of the missense mutants Y236S-Syn II and G464R-hSyn II in neurons nucleofected at early stages of development with ires-Tomato constructs (Fig. 2A, right) or in HeLa cells transfected with constructs coding for fusion proteins with mCherry (Fig. 2B) elicited expression levels that were fully comparable with those of WT*-*hSyn II.

Confocal imaging of Hela cells transfected with mCherry-tagged variants of either WT-hSyn II, A94fs199X-hSyn II, Y236S-hSyn II or G464R-hSyn II fully confirmed these results (Fig. 2C): WT-hSyn II and its missense variants were expressed in the cytoplasm of transfected cells, whereas A94fs199X-hSyn II was virtually absent. The practically complete lack of expression of the A94fs199X-hSyn II mutant after cDNA transfection could be attributable to poor translation efficiency and/or fast degradation of the aberrant protein.

### Hsyn II missense mutants are unable to rescue the *SYN2* KO presynaptic phenotype

We next evaluated the impact of the two ASD-linked missense mutations on presynaptic function. To this aim, we performed dynamic imaging of exo-endocytosis by assessing the effects of the expression of the WT or the missense *SYN2* mutants at the level of single synaptic boutons. Based on ultrastructural investigations and immunoblotting with SV-specific markers (27,41), it has previously been reported that *SYN2* KO neurons display a reduced population of SVs, consistent with the intense depression observed in response to high-frequency stimulation (27). However, no data are available on the dynamics of the exo-endocytotic cycle of SVs and on SV trafficking in these neurons.

**Figure 3.**
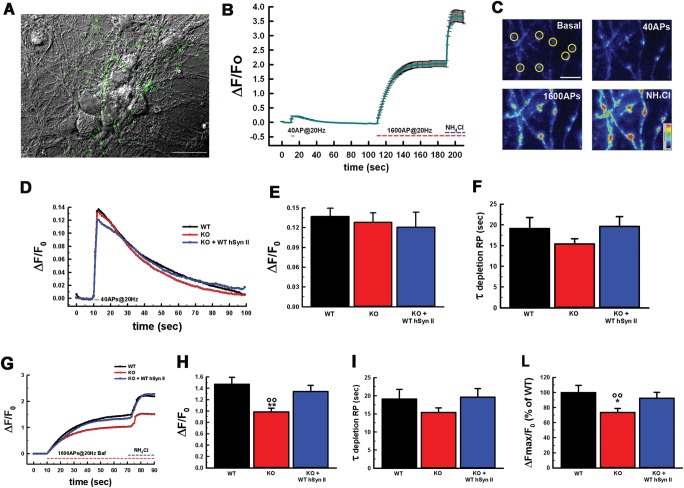
Expression of WT hSyn II rescues the impairment in the RP of SVs in *SYN2* KO neurons. (**A**) Representative image of WT hippocampal primary neurons transfected with Syphy to evaluate the size and dynamics of SV pools. The image shows the fluorescent processes of a neuron transfected with SypHy (green) together with other non-transfected neurons visualized in phase contrast. Scale bar, 50 µm. (**B**) Changes in SypHy fluorescence intensity (means ± SEM) as a function of time recorded from a WT neuron. Electrical field stimulation with 40 AP at 20 Hz, followed by 1600 AP at 20 Hz in the presence of 1 µM bafilomycin and a final perfusion with 50 mm of NH_4_Cl were used to evaluate the RRP, the RP and the total SV content, respectively. (**C**) Representative intensity profile images of SypHy-expressing WT neurons at 17 DIV, showing SypHy fluorescence in axonal branches at rest (Basal) and in response to stimulations. Scale bar, 5 µm; look-up table rainbow. (**D–F**) Evaluation of the RRP. Ensemble averages of individual time courses of SypHy fluorescence in response to 40 AP at 20 Hz field stimulation (D) in WT (*n* = 20, black trace), *SYN2* KO (*n* = 21, red trace) and *SYN2* KO neurons reconstituted with WT-hSyn II (*n* = 10, blue trace). Mean (± SEM) RRP size calculated as the peak level of fractional fluorescence increase over resting levels (Δ*F*/*F*_0_; E) and mean (± SEM) time constant of endocytosis (*τ*; E) evaluated by fitting the fluorescence decay after stimulation by a single exponential function. (**G–L**) Evaluation of the RP and total SV content. Ensemble averages of individual time courses of SypHy fluorescence in response to stimulation with 1600 AP at 20 Hz in the presence of 1 μM bafilomycin (G) in WT (*n* = 20, black trace), *SYN2* KO (*n* = 21, red trace) and *SYN2* KO neurons re-expressing WT-hSyn II (*n* = 10, blue trace). Mean (± SEM) RP size evaluated as Δ*F*/*F*_0_ (H) and mean (± SEM) time constant of RP depletion (*τ*; I) evaluated by fitting the fluorescence increase during stimulation by a single exponential rise. The total SypHy fluorescence was calculated as the fractional fluorescence plateau reached after NH_4_Cl over basal fluorescence (ΔF_MAX_/F_0_; L). Data (means±SEM) are expressed in percent of the WT mean value. **P < 0.01 versus WT cells; **P < 0.01 versus *SYN2* KO-expressing WT-hSyn II; one-way ANOVA on Ranks (Kruskal–Wallis test) followed by the Dunn's test. Experiments were performed from *n* = 3 independent preparations, with 12–20 boutons analyzed for each experiment.

Thus, before testing the biological activity of the Syn II mutants, it was necessary to study the *SYN2* KO phenotype and whether the potential impairments in SV dynamics could be rescued by expression of the WT-hSyn II. WT hippocampal neurons, *SYN2* KO neurons and *SYN2* KO neurons expressing the mCherry variant of hSyn II were transfected with synaptophysin-pHluorin (SypHy), a chimeric SV probe whose fluorescence is low in the acidic intravesicular environment and strongly increases when exposed to the extracellular medium during exocytosis (49–51). To evaluate the effect of *SYN2* deletion and hSyn II reconstitution on SV trafficking evoked by electrical activity, stimulation protocols triggering action potentials (APs) were applied that allowed to estimate the size of either the RRP (40 APs at 20 Hz) or the RP (1600 APs at 20 Hz in the presence of bafilomycin), followed by exposure to 50 mm of the alkaline agent NH_4_Cl to reveal the total SV pool that also includes release-reluctant SVs [(52); Fig. 3A–C].

The size of the RRP and the kinetics of endocytosis were not significantly affected in *SYN2* KO neurons (Fig. 3D–F), which is consistent with a substantially preserved number of morphologically docked SVs in *SYN2* KO terminals (41). In contrast, the size of the RP was markedly decreased in *SYN2* KO neurons with only a slight and non-significant increase of its depletion rate (Fig. 3G–I). Interestingly, the expression of hSyn II was able to fully rescue the *SYN2* KO phenotype and returned the RP size and time constant of depletion to the levels observed in WT neurons (Fig. 3G–I). In agreement with previous observations obtained with different methodologies, the total content of SVs, evaluated as Δ*F*_MAX_/*F*_0_ was significantly impaired in *SYN2* KO terminals with respect to WT terminals, and was virtually normalized upon reconstitution of WT-hSyn II (Fig. 3L).

**Figure 4.**
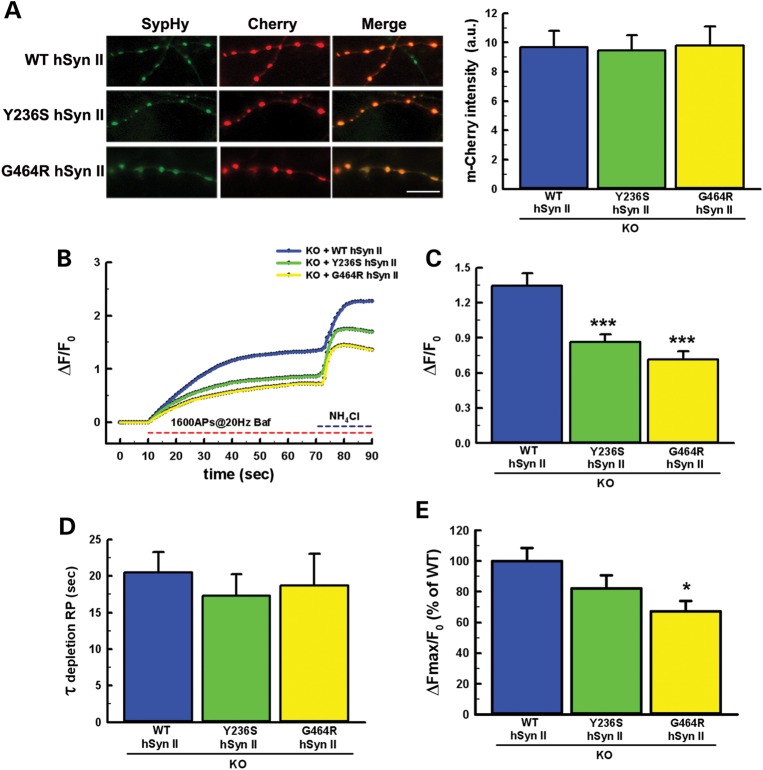
hSyn II missense mutants target to nerve terminals, but are unable to rescue the *SYN2* KO phenotype. (**A**) Synaptic expression of mCherry-tagged hSyn II variants. *Left:* representative images of SypHy (green) and either WT-hSyn II, Y236S-hSyn II or G464R-hSyn II tagged with mCherry (red) in *SYN2* KO hippocampal neurons transfected at 14 DIV and analyzed at 17 DIV. Merge panels show that all hSyn II variants display a virtually complete colocalization at SypHy-positive synaptic puncta. Scale bar, 20 µm. *Right:* Quantification of the presynaptic expression of mCherry-tagged WT-hSyn II, Y236S-hSyn II and G464R-hSyn II in *SYN2* KO neurons. The expression of WT and mutant mCherry-labeled hSyn II was analyzed by quantifying the mCherry fluorescence intensity at stimulation-responsive SypHy-positive puncta. Data are means ± SEM of *n* = 10 for WT-hSyn II, *n* = 10 for Y236S-hSyn II and *n* = 6 for G464R-hSyn II. The point mutations in hSyn II did not affect the targeting of the exogenous protein to nerve terminals. (**B**) Averaged ensembles of individual time courses of SypHy fluorescence dequenching in response to field stimulation (1600 APs at 20 Hz, 1 µM bafilomycin) followed by 50 mm NH_4_Cl in *SYN2* KO neurons co-transfected with SypHy and either WT-hSyn II (blue trace; *n* = 10), Y236S-hSyn II (green trace; *n* = 10) or G464R-hSyn II (yellow trace; *n* = 6). ROIs of 1.7 µm diameter corresponding to responsive synaptic boutons were manually selected in a pretrial round and analyzed for fluorescence intensity changes versus time. 15–20 ROIs were analyzed per experiment. SypHy dequenching was calculated as fractional fluorescence increase over resting levels (Δ*F*/*F*_0_; means ± SEM). (**C–E**) Quantitative analysis of RP and total SV content. RP size evaluated as Δ*F*/*F*_0_ plateau values (C), time constant of RP depletion (*τ*; D) and total SypHy fluorescence calculated as ΔF_MAX_/F_0_ and expressed in percentage of WT-hSyn II (E). Data are means ± SEM from the same experiments shown in (B). For further details, see legend to Figure 3 and the section Materials and Methods. *P < 0.05; ***P < 0.001 versus WT-hSyn II, ANOVA on ranks (Kruskal–Wallis test) followed by Dunn's method.

We next used the same experimental system with the missense mutants to determine their ability to rescue the phenotype when compared with WT-hSyn II. However, before a presynaptic effect of the mutants could be studied, it was necessary to demonstrate that, albeit normally expressed (Fig. 2), the hSyn II mutants were also correctly targeted to nerve terminals to exert their physiological actions. Thus, we expressed mCherry chimeras of WT-hSyn II, Y236S-hSyn II or G464R-hSyn II in *SYN2* KO hippocampal neurons that were co-transfected with SypHy and measured the mCherry fluorescence intensity at SypHy-positive puncta in which the SypHy fluorescence was dynamically regulated by activity. As shown in Figure 4A, both hSyn II mutants were correctly targeted to nerve terminals and their presynaptic levels were indistinguishable from those of WT-hSyn II.

Finally, we analyzed whether the expression of either hSyn II missense mutant was able to rescue the impairment in the RP size observed after constitutive deletion of endogenous Syn II as effectively as the WT-hSyn II did. As shown in Figure 4B and C, either mutant was unable to correct the *SYN2* KO phenotype and the RP size remained at the same *SYN2* KO level, markedly and significantly lower than the level reached after rescue with WT-hSyn II. No detectable effects were observed on the kinetics of RP mobilization that was not significantly altered in the *SYN2* KO neurons (Figs 3I and 4D). The Y236S and G464R mutants were also less effective than WT-hSyn II in rescuing the total SV content of *SYN2* KO terminals, with a clear-cut and significant loss-of-function for the G464R mutant (Fig. 4E).

### The G464R-hSyn II mutant impairs axon outgrowth and dendritic branching in *SYN2* KO hippocampal neurons at early stages of *in vitro* development

**Figure 5.**
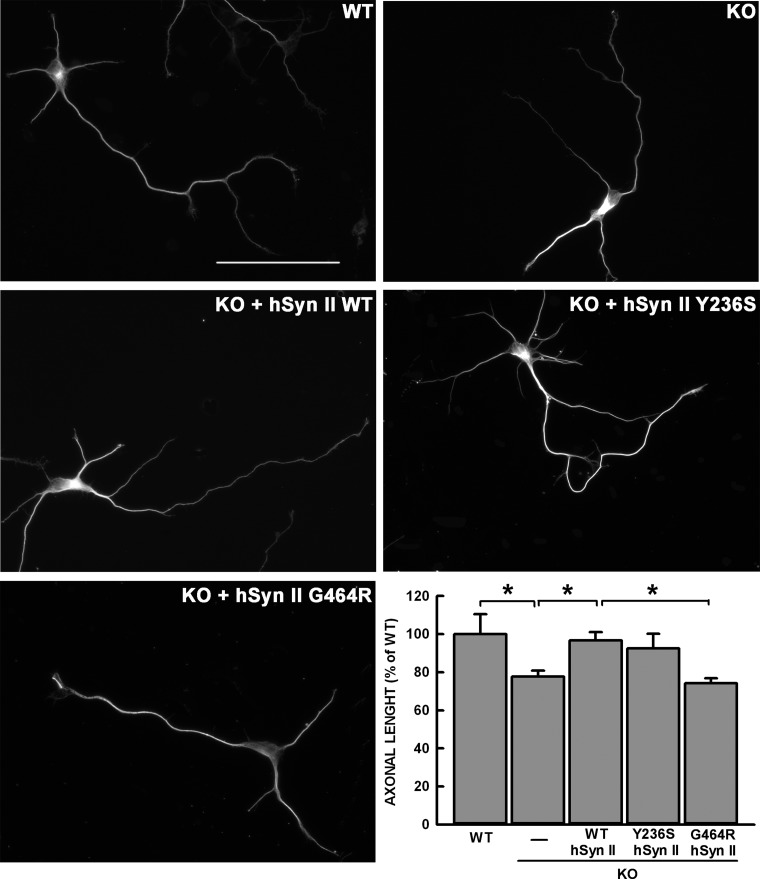
The G464R-hSyn II mutant impairs early stages of axon outgrowth. Representative images of WT and *SYN2* KO hippocampal neurons transfected at 0 DIV with either pCAGGS-ires-Tomato vector alternatively encoding for WT-hSyn II, G464R-hSyn II, Y236S-Syn II isoforms or with the vector alone (KO). β-tubulin staining of Tomato-positive neurons is shown. Scale bar, 50 μm. Identified axons at 3 DIV were measured by ImageJ tracing. Within each experimental session, the length of the axons measured in Syn II-transfected neurons was normalized to the average length of the axons measured in WT neurons. The plot shows the mean length values (± SEM) of axons calculated from *n* = 62 WT neurons, *n* = 76 *SYN2* KO neurons, *n* = 98 *SYN2* KO neurons expressing WT-hSyn II, *n* = 42 *SYN2* KO neurons expressing Y236S-hSyn II and *n* = 59 *SYN2* KO neurons expressing G464R-hSyn II, from *n* = 6 independent experiments. Statistical analysis was performed by one-way ANOVA followed by the Bonferroni's multiple comparisons test. *P < 0.05 as indicated.

**Figure 6.**
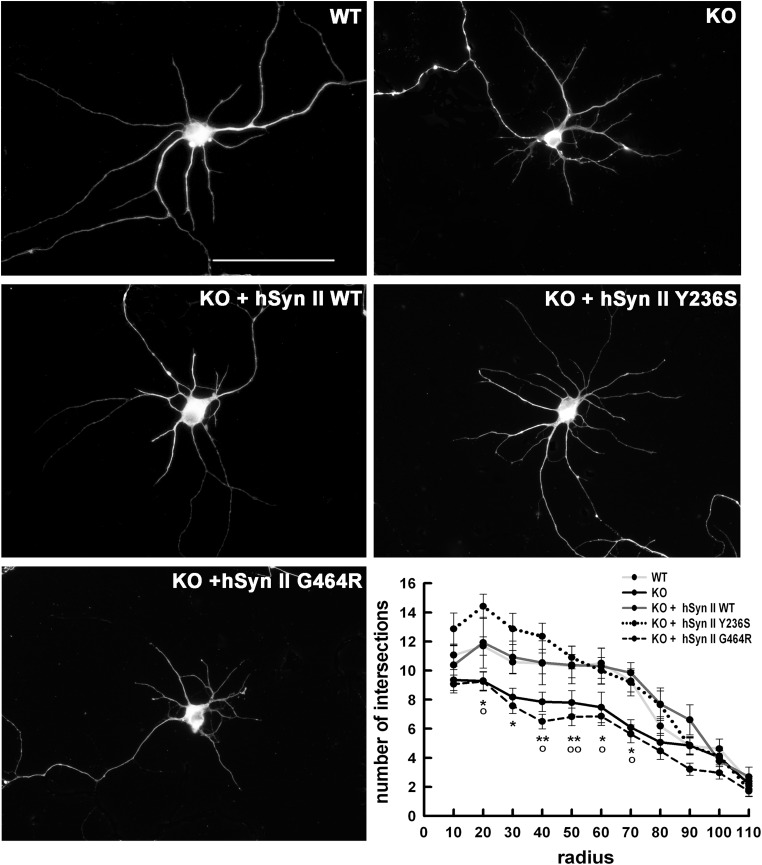
The G464R-hSyn II mutant impairs dendritic branching in *SYN2* KO hippocampal neurons at early stages of *in vitro* development. Representative images of WT and *SYN2* KO hippocampal neurons transfected at 4 DIV with either pCAGGS-ires-Tomato vector alternatively encoding for WT-hSyn II, Y236S-hSyn II and G464R-hSyn II or with Tomato vector alone (KO). β-tubulin staining of Tomato-positive neurons is shown. Scale bar, 50 μm. Dendritic arborization at 7 DIV was measured by Sholl analysis, counting the number of intersections of dendrites with concentric circles centered to the cell body and whose radius was increased at regular steps of 10 μm. Quantitative analysis of dendritic arborization was calculated from *n* = 31 WT neurons (light gray line), *n* = 58 *SYN2* KO neurons (black line) and *n* = 67, 31 and 34 *SYN2* KO neurons expressing WT-hSyn II (dark gray line), Y236S-hSyn II (dotted line) or G464R-hSyn II (broken line), respectively, from *n* = 3 independent experiments. Data, expressed as means ± SEM, were analyzed using one-way ANOVA followed by the Bonferroni's multiple comparison test. *P < 0.05; **P < 0.01 G464R-hSyn II versus WT-hSyn II; °*P* < 0.05; °°*P* < 0.01 *SYN2* KO neurons versus WT neurons or *SYN2* KO neurons reconstituted with WT-hSyn II.

Syn II expression peaks with synaptogenesis and remains elevated in mature neurons. However, the protein has been shown to be involved also in the early stages of neuronal development. Indeed, *SYN2* KO neurons display delayed and abnormal neurite outgrowth (38). While the A94fs199X-hSyn II mutant, due to the lack of expression, implies a full loss-of-function, the ability of the missense *SYN2* mutants to rescue the developmental phenotype of *SYN2* KO neurons is testable. To this aim, we nucleofected primary hippocampal neurons at 0 days *in vitro* (DIV) and followed the emergence and elongation of the axon as well as the development of the dendritic tree over the early developmental stages *in vitro* (Figs 5 and 6). As expected, *SYN2* KO neurons displayed a significant impairment in axon outgrowth at 3 DIV. Interestingly, this impairment was totally rescued by the expression of WT-hSyn II, demonstrating that mouse hippocampal neurons are a reliable model to assess the impact of mutations in neural development. When the two ASD-linked missense hSyn II mutants Y236S and G464R were expressed in place of the WT isoform, we found that the former promoted an increase in axon elongation that was indistinguishable from that of WT neurons, while the latter was virtually ineffective in rescuing the *SYN2* KO phenotype (Fig. 5).

A similar picture was observed when the development and complexity of the dendritic arborization was analyzed at 7 DIV by Sholl analysis (Fig. 6). Also in this case, the number of dendritic branchings was significantly decreased in *SYN2* KO neurons over a large range of *radii* from the cell body, and expression of either WT-hSyn II or its Y236S mutant was able to fully rescue the phenotype, bringing the number of branches to the levels observed in WT neurons. However, the expression of G464R-hSyn II was unable to correct the KO phenotype, indicating that this mutant, albeit normally expressed, exhibits a developmental loss-of-function.

## DISCUSSION

In addition to the several ‘synaptic’ ASD candidate genes identified in the last few years, including *NLGN3*, *NLGN4*, *SHANK2/3* and *IL1RAPL1* coding for postsynaptic proteins, as well as *NRXN1* and its homolog *CNTNAP2* and *RIMS3/NIM3*, coding for presynaptic proteins, we have recently reported that nonsense and missense mutations in *SYN1* (p.Q555X; p.A51G, p.A550 T and p.T567A) are associated with epilepsy and/or ASD (24,53), adding another presynaptic candidate gene as a common basis for these related diseases. Moreover, another nonsense mutation in *SYN1* (p.W356X), causing mRNA decay, was identified as the cause of epilepsy in a family with the history of syndromic epilepsy associated with behavioral disturbances, learning disabilities and one case of autism (25,54).

By screening a cohort of ASD individuals affected by ASD and/or epilepsy for *SYN* genes other than *SYN1*, we have identified several rare variants in *SYN2*, namely one nonsense (*n* = 1) and two missense (*n* = 2) mutations in ASD patients and one missense (*n* = 1) mutation in an epileptic patient. While the association did not reach the significance level for epilepsy, the rare variants were significantly associated with ASD. Both the ASD-linked nonsense and missense mutations in *SYN2* led to a loss of function being either non-expressed (frameshift/nonsense) or expressed and targeted to nerve terminals, but inactive in rescuing the *SYN2* KO phenotype (missense). The identification of *SYN2* as a predisposing gene for non-syndromic ASD further reinforces the notion that the *SYN* gene family is an important component of the ‘synaptic ASD pathway’. Interestingly, all the ASD individuals with *SYN2* mutations described here are males (*n* = 3) and the mutant alleles have been transmitted by unaffected females. This observation, although on a limited number of individuals, is consistent with the recent report on the autosomal *SHANK1* gene deletions that have been associated with ASD in males, but not in females (55). The occurrence of ASD only in males with *SHANK1* and *SYN2* would thus represent an example of autosomal sex-limited expression. The mechanism underlying this higher penetrance in males remains to be determined (56), although these observations are consistent with the ‘protective factor’ model, suggesting that there is a higher threshold in females in order to develop ASD (57). Autosomal sex-limited expression, in addition to rare mutations in X-linked genes (e.g. *SYN1*, *NLGN3* and *NRLGN4*, *IL1RAPL1*, *PTCHD1*), may thus contribute to the increased prevalence of ASD in males when compared with females (5,18,24,58).

Previous studies have suggested an association of *SYN2* rs3773364 A>G polymorphism with febrile seizures in the UK, Irish and Finnish cohorts [EPIGEN Epilepsy Genetic Consortium; (47)] and in Indian patients with idiopathic epilepsy (48), but not in the Australian cohort (47) or in Malaysian epileptic patients (59). Moreover, a positive association between schizophrenia and variants of the *SYN2* gene, such as SNPs and insertion/deletion polymorphisms, has been observed in Chinese subjects (60,61), in the Korean population (62) and in northern European families (63). Interestingly, these genetic studies, together with the rare variants described here, are reflected in the phenotype of *SYN2* KO mice. These mice are epileptic from 2 to 3 months of age and display behavioral abnormalities that are reminiscent of deficits observed in other known animal models of intellectual disability, schizophrenia and ASD (27,34,42–44,46,64). We have recently shown that the deletion of Syn isoforms widely affects social and repetitive behaviors, resulting in ASD-related phenotypes. In particular, *SYN2* deletion causes the most severe phenotype with respect to the deletion of other Syn isoforms, with extensively impaired social behavior and memory, altered exploration of a novel environment and increased self-grooming (46).

Syn II shares with Syn I a large NH_2_-terminal domain and the property of being phosphorylated by several kinases (PKA, CaMKI/IV, MAPK/Erk and Src), but diverges in its C-terminal domain (31). Similarly to Syn I, Syn II is specifically associated with the cytoplasmic membrane of SVs and interacts with actin filaments in a phosphorylation-dependent manner, although its interactions appear stronger than those of Syn I (65). While both isoforms are thought to control the density of SVs at the nerve terminal and regulate their availability for release, they display non-overlapping roles in nerve terminal function. We recently found that, although the total nerve terminal SV complement is markedly decreased in both *SYN1* and *SYN2* KO mice (28,41,53), the resulting effects on synaptic transmission are isoform specific. The lack of either isoform has distinct effects on SV pool size and dynamics. While *SYN1* deletion decreases both the RRP and the RP (24,36,66), deletion of *SYN2* only impairs the RP, leaving the RRP virtually unaffected (41). Moreover, synaptic depression is more intense in *SYN2* KO mice (27). In addition, we recently showed that Syn I and Syn II play opposite roles in regulating the release dynamics of inhibitory neurons, with Syn II facilitating the delayed asynchronous release of GABA and contributing to the extent of tonic inhibition, and Syn I enhancing the synchronous release response versus the asynchronous one (37,41).

The dynamic analysis of SV exo-endocytosis with SypHy fluorescence revealed that *SYN2* deletion does not affect the size of the RRP or the kinetics of endocytosis, but specifically decreases the size of the RP and the total SV population without major effects on the kinetics of SV depletion. These observations are consistent with: (i) the decreased expression of SV proteins (but not of synapse density or pre- and post-synaptic proteins) observed in *SYN2* KO brains (27); (ii) the decreased overall SV density together with a substantially preserved number of SVs docked to the active zone observed by ultrastructural studies of *SYN2* KO central and peripheral synapses (41,67); (iii) the pivotal role of the Syn IIa isoform in controlling the size of the RP of SVs, being the only Syn isoform capable of rescuing synaptic depression in *SYN1/2/3* triple KO mice (68); (iv) the unaffected RRP size upon expression of SynIIa in *SYN1/2/3* triple KO central synapses under physiological Ca^2+^ conditions (68). The latter finding is not in agreement with the observation that microinjection of a truncated form of recombinant SynII into SynII KO neuromuscular junctions reduced the number of docked SVs under low Ca^2+^ conditions (69). However, the substantial differences in synapse type, extracellular Ca^2+^ levels and type of expressed protein do not allow a direct comparison. Overall, these data underline the primary effects of Syn II in the formation and maintenance of SV pools in nerve terminals.

Before studying the impact of *SYN2* mutations on SV trafficking and neural development, we checked whether the expression of the homologous hSyn II was able to efficiently rescue the synaptic and developmental phenotypes of mouse *SYN2* KO neurons (27,34,38). The full rescue of SV pool size, axon outgrowth and neuropil complexity by the human ortholog confirmed the relevance of our experimental system.

Except for the A94fs-hSyn II mutant that was not translated in either HeLa cells or primary neurons, we found that the missense hSyn II mutants are expressed and targeted to nerve terminals in *SYN2* KO neurons. However, both missense hSyn II mutants displayed a clear loss-of-function when challenged with the rescue of the *SYN2* KO presynaptic phenotype and were both unable to correct the impaired RP size and total SV content that were virtually normalized by the expression of WT-hSyn II.

In neuronal development, Syn II plays an important role in regulating the early steps of neurite outgrowth and synapse formation, without affecting the number of synapses at the end of synaptogenesis (38). In contrast to Syn I, the Syn II-linked developmental phenotype also affects dendrite arborization, an effect that is likely to be secondary to the more marked delay in development displayed by *SYN2* KO neurons. This indicates that Syn II has a more profound structural role compared with Syn I, likely attributable to the stronger interactions of the former isoform with the cytoskeleton (65). With respect to WT-hSyn II, the G464R mutation predicted to be deleterious was totally unable to rescue the developmental KO phenotype, while the Y236S mutation, predicted to have a milder impact on Syn II function, was similar to WT-hSyn II.

The SYN2 gene encodes for both hSynIIa and hSynIIb. While the A94fs199X and Y236S mutations affect both SynIIa and SynIIb isoforms, the G464R mutation affects SynIIa only. Although SynIIb was reported to have a role in synaptogenesis in neuroblastoma × glioma cells (32), we only concentrated on the hSynIIa isoform for the following reasons: (i) the hSynIIa isoform is involved in all mutations and therefore represented the only means to directly compare the effects of the three mutations; (ii) SynIIa was the only Syn isoform able to rescue the synaptic depression phenotype of *SYN1/2/3* triple KO neurons, while SynIIb had a much weaker effect (68) and (iii) hSynIIa was able to fully rescue both the developmental and the synaptic SynII KO phenotype under our experimental conditions.

While the hSyn II mutants described here displayed a predominant loss-of-function character in primary *SYN2* KO neurons, their pathogenic role in patients in whom only one allele is mutated could result from either a haploinsufficiency with impaired Syn II expression or a dominant-negative effect towards the endogenous Syn II. The fact that the G464R mutation affecting only SynIIa, but not SynIIb, had a stronger impact on neuronal development compared with the Y236S-hSyn II mutant further indicates that SynIIa may be sufficient to perform all functions of the SYN2 gene, as previously reported (68). The stronger effect the G464R mutant could also be ascribed to the appearance of a new canonical Class II SH3 binding motif (xPxxPxR/K) by the R substitution in position 464 of SynIIa. The uncovering of a novel SH3-binding site could activate anomalous interactions with Src, PI3 K or proteins involved in SV cycling, with potential impact on the molecular interactions and adhesive properties of the mutant protein.

The strong association between epilepsy and ASD suggest that common mechanisms of synaptic dysfunction may underlie both diseases. Syn II is not essential for exocytosis, but is important for synapse maturation and remodeling. ASD manifestations begin in the second to third year of life, a period of intense refinement, remodeling and experience-dependent plasticity of synapses. This period fully overlaps with the developmental expression pattern of Syn IIa/IIb that peaks at 1–3 months after birth (70). In the healthy brain, a balance of excitation and inhibition is essential for all functions, from physiological network activity and oscillations to cognitive processes. Based on mouse KO studies, disruption of Syn isoforms is associated with an altered excitatory/inhibitory balance with serious consequences in postsynaptic integration, network computation, excitability and activity-dependent plasticity. Interestingly, dysfunctions of GABAergic signaling are strongly associated with the behavioral deficits observed in ASD patients (71). Hence, in addition to *SYN1*, also *SYN2* can participate in the ‘synaptic autism pathway’, implicating the whole Syn family of synaptic genes essential for activity-dependent changes in neuronal function in the pathogenesis of ASD.

## MATERIALS AND METHODS

### Characterization of epilepsy and ASD phenotypes

Affected individuals gave informed consent, and the study was approved by the ethics committee of the CHUM. Clinical evaluations for the epilepsy phenotype included interview with patients and relatives, standard EEG and brain MRI. Seizure and syndrome classification were made according to the International League Against Epilepsy (1989). In the affected individual bearing the P253A mutation, the clinical phenotype consisted of complex partial seizures. In this individual, EEG did not reveal interictal epileptic activity, and brain MRI was unremarkable. Otherwise, all the affected individuals from our cohort exhibited clinical features compatible with idiopathic partial epilepsy. Standardized Assessment for Diagnosis of Autism (ADI-R and/or ADOS-G Module-3) was performed in our ASD cohort, as previously described (11). Neuropsychological assessment included the WAIS III and WISC Q5 IV in some individuals. Psychological and psychiatric assessments were performed by neuropsychologists and psychiatrists blinded to the genetic status (Table 2; see also Supplementary Material, Information).

### Sequencing of SYN2 gene

The genomic organization of the human *SYN2* gene was determined by aligning sequences from *SYN2* mRNA (Genbank NM_133625) to the corresponding genomic sequence on chromosome 3 (Genbank NC_000003.11). Primers were designed to amplify 400–600 bp fragments from genomic DNA to screen all coding portions of the gene. Portions of the *SYN2* gene were amplified by polymerase chain reaction (PCR) and analyzed by direct sequencing on an automatic sequencer (ABI3730; Applied Biosystems). Due to technical difficulties, the first exon was not sequenced. Primer sequences are available upon request.

### Cloning of SYN2 and mutagenesis

Primers were designed to amplify by PCR the complete open reading frame of hSyn IIa from a human brain cDNA library (Marathon-ready; Clontech). The PCR products were subcloned into either pCAGGS-ires-Tomato or pmCherryC1 (Clontech) vector. The *SYN2* mutants were generated from WT-hSyn II cDNA, using site-directed mutagenesis (Quikchange II kit; Stratagene). Primer sequences are available upon request.

### Expression of hSyn II in HeLa cells

HeLa cells were cultured in Dulbecco's MEM (DMEM; Gibco) supplemented with 10% fetal bovine serum (Gibco), 1% penicillin–streptomycin (Gibco) and maintained at 37°C in a 5% CO_2_ humidified atmosphere. The day before transfection 3.5 × 10^5^ cells were plated on 60 mm plates. The medium was replaced with OPTIMEM medium (Gibco) 1 h before the transfection. Cells were co-transfected with 1.5 μg of pCAGGS-ires-Tomato or mCherry-tagged WT or mutated hSyn II cDNA and 2.5 μl of Lipofectamine 2000 reagent (Invitrogen) in OPTIMEM medium. After 4 h of incubation with the transfection mix, the medium was replaced with fresh DMEM and the cells were incubated under standard growth conditions for 48 h and then processed for western blotting or immunocytochemistry.

### Expression of hSyn II in primary hippocampal neurons

Homozygous *SYN2* KO mice and C57BL/6J WT littermates were used in accordance with the guidelines established by the European Communities Council (Directive 2010/63/EU of 22 September 2010) and were approved by the Italian Ministry of Health. Hippocampal neurons were prepared from WT and *SYN2* KO E17–18 mouse embryos, plated onto poly-l-lysine coated coverslips (Sigma) at 6 × 10^4^ cells/coverslip and maintained in a culture medium consisting of Neurobasal (Gibco) plus B-27 (Gibco, 1:50 v/v), Glutamax (Gibco, 1% w/v), penicillin–streptomycin (Gibco, 1%) at 37°C in a 5% CO_2_ humidified atmosphere. Before plating, neurons (1.5–4 × 10^6^) were nucleofected with Amaxa basal nucleofector kit for primary neurons (Lonza) and 4 μg of plasmid DNA (pCAGGS-ires-Tomato hSyn II) in the Amaxa nucleofector device according to manufacturer's protocol. Cells were incubated under standard growth conditions until 3 DIV and then processed for analysis of axon elongation or western blotting. For Sholl analysis, neurons were transfected at 4 DIV and fixed at 7 DIV (0.6 μg of cDNA and 6 μl of lipofectamine 2000 reagent; Invitrogen). For functional experiments, neurons were double transfected at 14 DIV with superecliptic SypHy and mCherry-tagged hSyn II, using 0.6 μg of cDNA and 6 μl of lipofectamine 2000 reagent (Invitrogen), diluted in Neurobasal medium without antibiotics, for each coverslip. Live imaging experiments were performed between 16 and 19 DIV.

### Western blotting

Total cell lysates were obtained from HeLa cells 48 h after transfection or from hippocampal neuron cultures at 7 DIV. Cells were lysed in lysis buffer (150 mm NaCl, 50 mm Tris, NP-40 1%, sodium dodecyl sulfate (SDS) 0.1%) supplemented with 1 mm PMSF/1 mm Pepstatin (Sigma). After 20 min of incubation, lysates were collected and clarified by centrifugation (10 min at 10 000*g*). The protein concentration of samples was estimated with BCA (Pierce) and equivalent amounts of protein were subjected to SDS-polyacrylamide gel electrophoresis on 10% polyacrylamide Mini-PROTEAN TGX precast gels (BioRad) according to the manufacturer's instructions. Gels were then blotted onto nitrocellulose membranes (Whatman). After a brief staining of the blot with 0.1% Ponceau S., membranes were blocked for 1 h in 5% milk in Tris-buffered saline (10 mm Tris, 150 mm NaCl, pH 8.0) plus 0.1% Triton X-100 and incubated overnight at 4°C with the following primary antibodies: anti-actin (1:5000, Sigma), anti-Syn II monoclonal 19.21 (1:1000; ref. 72) and anti-Cherry (1:1000, Clontech). Membranes were washed and incubated for 1 h at room temperature with peroxidase-conjugated anti-mouse (1:3000; BioRad) or anti-rabbit (1:5000; BioRad) antibodies. Bands were revealed with the ECL chemiluminescence detection system (Thermo Scientific).

### Immunocytochemistry

Primary hippocampal neurons were fixed with 4% paraformaldehyde, 4% sucrose in phosphate-buffered saline (PBS), pH 7.4. After several washes in PBS, cells were permeabilized with 0.1% Triton X-100 in PBS for 5 min and blocked with 0.1% Triton X-100, 4% fetal bovine serum in PBS for 30 min. Samples were sequentially incubated with primary antibodies (anti-β-tubulin from Sigma diluted 1:800; anti-panaxonal neurofilament SMI-312 from Covance diluted 1:1,000; anti-Syn II monoclonal 19.21 diluted 1:1000) in blocking solution (3 h at room temperature or overnight at 4°C), followed by Alexa 488-conjugated secondary antibodies (Invitrogen; 1:500 for 1 h at room temperature). The F-actin cytoskeleton was stained by incubation with AlexaFluor-488 phalloidin (Invitrogen; diluted 1:500) for 1 h. After several washes in PBS, coverslips were mounted using Prolong Gold antifade reagent (Invitrogen) containing DAPI (4′,6′-diamidino-2-phenylindole) for nuclear staining. Images were collected on either an Olympus IX-81 microscope with an MT20 Arc/Xe lamp, 40× objective, using the Excellence RT software (Olympus, Hamburg, Germany) or a Leica TCS SP5 AOBS TANDEM confocal microscope, 40×/0.80 APO L W UVI objective, using the Leica LAS AF software, and analyzed with ImageJ software.

### Morphometric analysis

Hippocampal neurons were either nucleofected at 0 DIV and fixed at 3 DIV to analyze axon outgrowth, or transfected at 4 DIV and fixed at 7 DIV to analyze dendritic arborization. A retrospective β-tubulin staining was performed to discriminate between glia and neuron cells. Axons at 3 DIV, identified as the longest process of each neuron with non-tapering morphology, were measured using the ImageJ software based on β-tubulin staining. The identity of the axons was retrospectively confirmed using SMI-312 staining. Dendritic arborization at 7 DIV was analyzed by Sholl analysis, counting the number of intersections of dendrites with concentric circles centered on the cell body whose radius was increasing at regular steps of 10 μm.

### Live-cell imaging assay

Optical recordings were performed at 16–19 DIV (i.e. 48–72 h after transfection) when a proper degree of maturation of the network was reached. Transfected hippocampal neurons, grown onto poly-l-lysine-treated glass coverslips, were transferred to a stimulation chamber (volume ∼100 μl; Warner Instruments, Hamden, CT, USA), immersed in Tyrode Solution (140 mm NaCl, 3 mm KCl, 2 mm CaCl_2_, 1 mm MgCl_2_, 10 mm HEPES-buffered to pH 7.4, 10 mm glucose) supplemented with glutamate receptor inhibitors (10 μM CNQX/50 μM APV) and positioned on the stage of an IX-81 motorized inverted epifluorescence microscope (Olympus). An MT20 Hg-Xe lamp (Olympus) was used as light source with 480 ± 20 nm excitation, 495 nm dichroic and 525 ± 50 nm emission filters to detect the SypHy signal and 560 ± 40 nm excitation, 585 nm dichroic and 630 ± 75 nm emission filters to detect the mCherry signal. One image of mCherry-hSyn II was acquired at the beginning of each experiment to verify the presence of the protein in the presynaptic boutons to be analyzed and used for the region-of-interest (ROI) selection. Time-lapse images were acquired at 1 Hz for 100 s with a Hamamatsu Orca-ER IEEE1394 CCD camera (Hamamatsu Photonics, Hamamatsu City, Japan) using a UplanSapo 60X1.35 NA oil-immersion objective (Olympus). Cells were maintained in a saline solution through a laminar-flow perfusion and the chosen field was stimulated after 10 s of baseline acquisition. APs were evoked by passing 1 ms current pulses, yielding fields of 10 V/cm, through platinum iridium electrodes using an AM 2100 stimulator (AM Systems, Carlsborg, WA). Images were analyzed using Excellence RT software (Olympus). Circular ROIs of 1.7 μm diameter were positioned at the center of manually selected individual puncta that were selected on the basis of: (i) a clear response to stimulation at 20 Hz (2 s) in a pretrial round and (ii) the concomitant expression of hSyn II in the red channel. Fluorescence time courses were analyzed with SigmaPlot 10.0 (Systat Software, Chicago, IL).

### Statistical analysis

The excess of rare variants found in ASD (*n* = 380) versus control (*n* = 670) chromosomes was assessed by using the Fisher's exact test. The two-sided *P*-value was considered. The normal distribution of experimental data was assessed using the Shapiro–Wilk test. Data with normal distribution were analyzed by one-way analysis of variance (ANOVA) followed by the *post hoc* Bonferroni's multiple comparison test. Non-normally distributed data were analyzed by ANOVA on ranks test (Kruskal–Wallis test) followed by Dunn's test. The statistical analysis was carried out using the Sigmaplot 12.0 software (Systat Software, Chicago, IL, USA). Significance level was preset to *P* < 0.05. Data are expressed as means ± SEM throughout for the number of cells (*n*).

## SUPPLEMENTARY MATERIAL

Supplementary Material is available at *HMG* online.

*Conflict of Interest statement*. None declared.

## FUNDING

This work was supported by grants from the Canadian Institute for Health Research and Genome Canada (to P.C. and G.A.R.), the Savoy Foundation (to L.P.), the Italian Ministry of University and Research (PRIN to A.C., F.B. and F.V.), the Italian Ministry of Health Progetto Giovani (to A.F.), the Compagnia di San Paolo, Torino (to A.F., F.V. and F.B.) and the Quebec Ministry of International Relationships and Italian Ministry of Foreign Affairs (to P.C. and F.B.). Funding to pay the Open Access publication charges for this article was provided by Telethon, Italy.

## Supplementary Material

Supplementary Data

## References

[1] Rapin I. (1997). Autism. N. Engl. J. Med..

[2] Jorde L.B., Hasstedt S.J., Ritvo E.R., Mason-Brothers A., Freeman B.J., Pingree C., McMahon W.M., Petersen B., Jenson W.R., Mo A. (1991). Complex segregation analysis of autism. Am. J. Hum. Genet..

[3] Steffenburg S., Gillberg C., Hellgren L., Andersson L., Gillberg I.C., Jakobsson G., Bohman M. (1989). A twin study of autism in Denmark, Finland, Iceland, Norway and Sweden. J. Child Psychol. Psychiatry.

[4] Bailey A., Le Couteur A., Gottesman I., Bolton P., Simonoff E., Yuzda E., Rutter M. (1995). Autism as a strongly genetic disorder: evidence from a British twin study. Psychol. Med..

[5] Jamain S., Quach H., Betancur C., Rastam M., Colineaux C., Gillberg I.C., Soderstrom H., Giros B., Leboyer M., Gillberg C. (2003). Mutations of the X-linked genes encoding neuroligins NLGN3 and NLGN4 are associated with autism. Nat. Genet..

[6] Laumonnier F., Bonnet-Brilhault F., Gomot M., Blanc R., David A., Moizard M.P., Raynaud M., Ronce N., Lemonnier E., Calvas P. (2004). X-linked mental retardation and autism are associated with a mutation in the NLGN4 gene, a member of the neuroligin family. Am. J. Hum. Genet..

[7] Yan J., Oliveira G., Coutinho A., Yang C., Feng J., Katz C., Sram J., Bockholt A., Jones I.R., Craddock N. (2005). Analysis of the neuroligin 3 and 4 genes in autism and other neuropsychiatric patients. Mol. Psychiatry.

[8] Zhang C., Milunsky J.M., Newton S., Ko J., Zhao G., Maher T.A., Tager-Flusberg H., Bolliger M.F., Carter A.S., Boucard A.A. (2009). A neuroligin-4 missense mutation associated with autism impairs neuroligin-4 folding and endoplasmic reticulum export. J. Neurosci..

[9] Durand C.M., Betancur C., Boeckers T.M., Bockmann J., Chaste P., Fauchereau F., Nygren G., Rastam M., Gillberg I.C., Anckarsater H. (2007). Mutations in the gene encoding the synaptic scaffolding protein SHANK3 are associated with autism spectrum disorders. Nat. Genet..

[10] Moessner R., Marshall C.R., Sutcliffe J.S., Skaug J., Pinto D., Vincent J., Zwaigenbaum L., Fernandez B., Roberts W., Szatmari P. (2007). Contribution of SHANK3 mutations to autism spectrum disorder. Am. J. Hum. Genet..

[11] Gauthier J., Bonnel A., St-Onge J., Karemera L., Laurent S., Mottron L., Fombonne E., Joober R., Rouleau G.A. (2005). NLGN3/NLGN4 Gene mutations are not responsible for autism in the Quebec population. Am. J. Med. Genet. B Neuropsychiatr. Genet..

[12] Kim H.G., Kishikawa S., Higgins A.W., Seong I.S., Donovan D.J., Shen Y., Lally E., Weiss L.A., Najm J., Kutsche K. (2008). Disruption of neurexin 1 associated with autism spectrum disorder. Am. J. Hum. Genet..

[13] Berkel S., Marshall C.R., Weiss B., Howe J., Roeth R., Moog U., Endris V., Roberts W., Szatmari P., Pinto D. (2010). Mutations in the SHANK2 synaptic scaffolding gene in autism spectrum disorder and mental retardation. Nat. Genet..

[14] Strauss K.A., Puffenberger E.G., Huentelman M.J., Gottlieb S., Dobrin S.E., Parod J.M., Stephan D.A., Morton D.H. (2006). Recessive symptomatic focal epilepsy and mutant contactin-associated protein-like 2. N. Engl. J. Med..

[15] Alarcon M., Abrahams B.S., Stone J.L., Duvall J.A., Perederiy J.V., Bomar J.M., Sebat J., Wigler M., Martin C.L., Ledbetter D.H. (2008). Linkage, association, and gene-expression analyses identify CNTNAP2 as an autism-susceptibility gene. Am. J. Hum. Genet..

[16] Arking D.E., Cutler D.J., Brune C.W., Teslovich T.M., West K., Ikeda M., Rea A., Guy M., Lin S., Cook E.H. (2008). A common genetic variant in the neurexin superfamily member CNTNAP2 increases familial risk of autism. Am. J. Hum. Genet..

[17] Bakkaloglu B., O'Roak B.J., Louvi A., Gupta A.R., Abelson J.F., Morgan T.M., Chawarska K., Klin A., Ercan-Sencicek A.G., Stillman A.A. (2008). Molecular cytogenetic analysis and resequencing of contactin associated protein-like 2 in autism spectrum disorders. Am. J. Hum. Genet..

[18] Piton A., Michaud J.L., Peng H., Aradhya S., Gauthier J., Mottron L., Champagne N., Lafreniere R.G., Hamdan F.F., Joober R. (2008). Mutations in the calcium-related gene IL1RAPL1 are associated with autism. Hum. Mol. Genet..

[19] Kumar R.A., Sudi J., Babatz T.D., Brune C.W., Oswald D., Yen M., Nowak N.J., Cook E.H., Christian S.L., Dobyns W.B. (2010). A de novo 1p34.2 microdeletion identifies the synaptic vesicle gene RIMS3 as a novel candidate for autism. J. Med. Genet..

[20] Walsh C.A., Morrow E.M., Rubenstein J.L. (2008). Autism and brain development. Cell.

[21] Bourgeron T. (2009). A synaptic trek to autism. Curr. Opin. Neurobiol..

[22] Tuchman R., Rapin I. (2002). Epilepsy in autism. Lancet Neurol..

[23] Dravet C. (2002). The behavioral disorders in epilepsy. Rev. Neurol. (Paris).

[24] Fassio A., Patry L., Congia S., Onofri F., Piton A., Gauthier J., Pozzi D., Messa M., Defranchi E., Fadda M. (2011). SYN1 loss-of-function mutations in autism and partial epilepsy cause impaired synaptic function. Hum. Mol. Genet..

[25] Garcia C.C., Blair H.J., Seager M., Coulthard A., Tennant S., Buddles M., Curtis A., Goodship J.A. (2004). Identification of a mutation in synapsin I, a synaptic vesicle protein, in a family with epilepsy. J. Med. Genet..

[26] Noebels J.L. (2003). Exploring new gene discoveries in idiopathic generalized epilepsy. Epilepsia.

[27] Rosahl T.W., Spillane D., Missler M., Herz J., Selig D.K., Wolff J.R., Hammer R.E., Malenka R.C., Sudhof T.C. (1995). Essential functions of synapsins I and II in synaptic vesicle regulation. Nature.

[28] Li L., Chin L.S., Shupliakov O., Brodin L., Sihra T.S., Hvalby O., Jensen V., Zheng D., McNamara J.O., Greengard P. (1995). Impairment of synaptic vesicle clustering and of synaptic transmission, and increased seizure propensity, in synapsin I-deficient mice. Proc. Natl. Acad. Sci. USA.

[29] Crowder K.M., Gunther J.M., Jones T.A., Hale B.D., Zhang H.Z., Peterson M.R., Scheller R.H., Chavkin C., Bajjalieh S.M. (1999). Abnormal neurotransmission in mice lacking synaptic vesicle protein 2A (SV2A). Proc. Natl Acad. Sci. USA.

[30] Janz R., Goda Y., Geppert M., Missler M., Sudhof T.C. (1999). SV2A And SV2B function as redundant Ca^2+^ regulators in neurotransmitter release. Neuron.

[31] Cesca F., Baldelli P., Valtorta F., Benfenati F. (2010). The synapsins: key actors of synapse function and plasticity. Prog. Neurobiol..

[32] Fornasiero E.F., Raimondi A., Guarnieri F.C., Orlando M., Fesce R., Benfenati F., Valtorta F. (2012). Synapsins contribute to the dynamic spatial organization of synaptic vesicles in an activity-dependent manner. J. Neurosci..

[33] Orenbuch A., Shalev L., Marra V., Sinai I., Lavy Y., Kahn J., Burden J.J., Staras K., Gitler D. (2012). Synapsin selectively controls the mobility of resting pool vesicles at hippocampal terminals. J. Neurosci..

[34] Fassio A., Raimondi A., Lignani G., Benfenati F., Baldelli P. (2011). Synapsins: from synapse to network hyperexcitability and epilepsy. Semin. Cell Dev. Biol..

[35] Gitler D., Takagishi Y., Feng J., Ren Y., Rodriguiz R.M., Wetsel W.C., Greengard P., Augustine G.J. (2004). Different presynaptic roles of synapsins at excitatory and inhibitory synapses. J. Neurosci..

[36] Chiappalone M., Casagrande S., Tedesco M., Valtorta F., Baldelli P., Martinoia S., Benfenati F. (2009). Opposite changes in glutamatergic and GABAergic transmission underlie the diffuse hyperexcitability of synapsin I-deficient cortical networks. Cereb. Cortex.

[37] Farisello P., Boido D., Nieus T., Medrihan L., Cesca F., Valtorta F., Baldelli P., Benfenati F. (2013). Synaptic and extrasynaptic origin of the excitation/inhibition imbalance in the hippocampus of synapsin I/II/III knockout mice. Cereb. Cortex.

[38] Fornasiero E.F., Bonanomi D., Benfenati F., Valtorta F. (2010). The role of synapsins in neuronal development. Cell Mol. Life Sci..

[39] Chin L.S., Li L., Ferreira A., Kosik K.S., Greengard P. (1995). Impairment of axonal development and of synaptogenesis in hippocampal neurons of synapsin I-deficient mice. Proc. Natl. Acad. Sci. USA.

[40] Feng J., Chi P., Blanpied T.A., Xu Y., Magarinos A.M., Ferreira A., Takahashi R.H., Kao H-T., McEwen B.S., Ryan T.A. (2002). Regulation of neurotransmitter release by synapsin III. J. Neurosci..

[41] Medrihan L., Cesca F., Raimondi A., Lignani G., Baldelli P., Benfenati F. (2013). Synapsin II desynchronizes neurotransmitter release at inhibitory synapses by interacting with presynaptic calcium channels. Nat. Commun..

[42] Corradi A., Zanardi A., Giacomini C., Onofri F., Valtorta F., Zoli M., Benfenati F. (2008). Synapsin-I- and synapsin-II-null mice display an increased age-dependent cognitive impairment. J. Cell Sci..

[43] Dyck B.A., Skoblenick K.J., Castellano J.M., Ki K., Thomas N., Mishra R.K. (2007). Synapsin II knockout mice show sensorimotor gating and behavioural abnormalities similar to those in the phencyclidine-induced preclinical animal model of schizophrenia. Schizophr. Res..

[44] Dyck B.A., Skoblenick K.J., Castellano J.M., Ki K., Thomas N., Mishra R.K. (2009). Behavioral abnormalities in synapsin II knockout mice implicate a causal factor in schizophrenia. Synapse.

[45] Porton B., Rodriguiz R.M., Phillips L.E., Gilbert J.W., Feng J., Greengard P., Kao H-T., Wetsel W.C. (2010). Mice lacking synapsin III show abnormalities in explicit memory and conditioned fear. Genes Brain Behav..

[46] Greco B., Managò F., Tucci V., Kao H.T., Valtorta F., Benfenati F. (2013). Autism-related behavioral abnormalities in synapsin knockout mice. Behav. Brain Res..

[47] Cavalleri G.L., Weale M.E., Shianna K.V., Singh R., Lynch J.M., Grinton B., Szoeke C., Murphy K., Kinirons P., O'Rourke D. (2007). Multicentre search for genetic susceptibility loci in sporadic epilepsy syndrome and seizure types: a case–control study. Lancet Neurol..

[48] Lakhan R., Kalita J., Misra U.K., Kumari R., Mittal B. (2010). Association of intronic polymorphism rs3773364 A>G in synapsin-2 gene with idiopathic epilepsy. Synapse.

[49] Miesenböck G., De Angelis D.A., Rothman J.E. (1998). Visualizing secretion and synaptic transmission with pH-sensitive green fluorescent proteins. Nature.

[50] Sankaranarayanan S., De Angelis D., Rothman J.E., Ryan T.A. (2000). The use of pHluorins for optical measurements of presynaptic activity. Biophys. J..

[51] Burrone J., Li Z., Murthy V.N. (2006). Studying vesicle cycling in presynaptic terminals using thegenetically encoded probe synaptopHluorin. Nat. Protoc..

[52] Fernandez-Alfonso T., Ryan T.A. (2008). A heterogeneous ‘resting’ pool of synaptic vesicles that is dynamically interchanged across boutons in mammalian CNS synapses. Brain Cell Biol..

[53] Lignani G., Raimondi A., Ferrea E., Rocchi A., Paonessa F., Cesca F., Orlando M., Tkatch T., Valtorta F., Cossette P. (2013). Epileptogenic Q555X SYN1 mutant triggers imbalances in release dynamics and short-term plasticity. Hum. Mol. Genet..

[54] Giannandrea M., Guarnieri F.C., Gehring N.H., Monzani E., Benfenati F., Kulozik A.E., Valtorta F. (2013). Nonsense-mediated mRNA decay and loss-of-function of the protein underlie the X-linked epilepsy associated with the W356X mutation in SYN1. PLoS ONE.

[55] Sato D., Lionel A.C., Leblond C.S., Prasad A., Pinto D., Walker S., O'Connor I., Russell C., Drmic I.E., Hamdan F.F. (2012). SHANK1 Deletions in males with autism spectrum disorder. Am. J. Hum. Genet.

[56] Baron-Cohen S., Lombardo M.V., Auyeung B., Ashwin E., Chakrabarti B., Knickmeyer R. (2011). Why are autism spectrum conditions more prevalent in males?. PLoS Biol..

[57] Szatmari P., Liu X.Q., Goldberg J., Zwaigenbaum L., Paterson A.D., Woodbury-Smith M., Georgiades S., Duku E., Thompson A. (2012). Sex differences in repetitive stereotyped behaviors in autism: implications for genetic liability. Am. J. Med. Genet. B. Neuropsychiatr. Genet..

[58] Noor A., Whibley A., Marshall C.R., Gianakopoulos P.J., Piton A., Carson A.R., Orlic-Milacic M., Lionel A.C., Sato D., Pinto D. (2010). Disruption at the PTCHD1 Locus on Xp22.11 in autism spectrum disorder and intellectual disability. Sci. Transl. Med..

[59] Haerian B.S., Lim K.S., Tan H.J., Wong C.P., Wong S.W., Tan C.T., Raymond A.A., Mohamed Z. (2011). Lack of association between synapsin II (SYN2) gene polymorphism and susceptibility epilepsy: a case-control study and meta-analysis. Synapse.

[60] Chen Q., He G., Qin W., Chen Q.Y., Zhao X.Z., Duan S.W., Liu X.M., Feng G.Y., Xu Y.F., St Clair D. (2004). Family-based association study of synapsin II and schizophrenia. Am. J. Hum. Genet..

[61] Chen Q., He G., Wang X.Y., Chen Q.Y., Liu X.M., Gu Z.Z., Liu J., Li K.Q., Wang S.J., Zhu S.M. (2004). Positive association between synapsin II and schizophrenia. Biol. Psychiatry.

[62] Lee H.J., Song J.Y., Kim J.W., Jin S.Y., Hong M.S., Park J.K., Chung J.H., Shibata H., Fukumaki Y. (2005). Association study of polymorphisms in synaptic vesicle-associated genes, SYN2 and CPLX2, with schizophrenia. Behav. Brain Funct..

[63] Saviouk V., Moreau M.P., Tereshchenko I.V., Brzustowicz L.M. (2007). Association of synapsin 2 with schizophrenia in families of northern european ancestry. Schizophr. Res..

[64] Etholm L., Bahonjic E., Walaas S.I., Kao H-T., Heggelund P. (2012). Neuroethologically delineated differences in the seizure behavior of synapsin 1 and synapsin 2 knock-out mice. Epilepsy Res..

[65] Nielander H.B., Onofri F., Schaeffer E., Menegon A., Fesce R., Valtorta F., Greengard P., Benfenati F. (1997). Phosphorylation-dependent effects of synapsin IIa on actin polymerization and network formation. Eur. J. Neurosci..

[66] Baldelli P., Fassio A., Valtorta F., Benfenati F. (2007). Lack of synapsin I reduces the readily releasable pool of synaptic vesicles at central inhibitory synapses. J. Neurosci..

[67] Samigullin D., Bill C.A., Coleman W.L., Bykhovskaia M. (2004). Regulation of transmitter release by synapsin II in mouse motor terminals. J. Physiol..

[68] Gitler D., Cheng Q., Greengard P., Augustine G.J. (2008). Synapsin IIa controls the reserve pool of glutamatergic synaptic vesicles. J. Neurosci..

[69] Coleman W.L., Bill C.A., Simsek-Duran F., Lonart G., Samigullin D., Bykhovskaia M. (2008). Synapsin II and calcium regulate vesicle docking and the cross-talk between vesicle pools at the mouse motor terminals. J. Physiol..

[70] Bogen I.L., Jensen V., Hvalby O., Walaas S.I. (2009). Synapsin-dependent development of glutamatergic synaptic vesicles and presynaptic plasticity in postnatal mouse brain. Neuroscience.

[71] Pizzarelli R., Cherubini E. (2011). Alterations of GABAergic signaling in autism spectrum disorders. Neural Plast..

[72] Vaccaro P., Dente L., Onofri F., Zucconi A., Martinelli S., Valtorta F., Greengard P., Cesareni G., Benfenati F. (1997). Anti-synapsin monoclonal antibodies: epitope mapping and inhibitory effects on phosphorylation and Grb2 binding. Brain Res. Mol. Brain Res..

