# Supplementary material for: SYN2 is an autism predisposing gene: loss-of-function mutations alter synaptic vesicle cycling and axon outgrowth
Source: Hum Mol Genet. 2013 Aug 15;23(1):90–103. doi: 10.1093/hmg/ddt401 (PMC3857945; doi:10.1093/hmg/ddt401)
Supplement: Supplementary Data [file supp_23_1_90__index.html]

SYN2 is an Autism Predisposing Gene: Loss-of-function Mutations Alter Synaptic Vesicle Cycling and Axon Outgrowth — SYN2 is an autism predisposing gene: loss-of-function mutations alter synaptic vesicle cycling and axon outgrowth — SYN2 is an autism predisposing gene: loss-of-function mutations alter synaptic vesicle cycling and axon outgrowth — Supplementary Data 

# *SYN2* is an autism predisposing gene: loss-of-function mutations alter synaptic vesicle cycling and axon outgrowth

## Supplementary Data

Supplementary Data

**Files in this Data Supplement:**

- Supplementary Data - Pdf file
