# Supplementary material for: SYN2 is an autism predisposing gene: loss-of-function mutations alter synaptic vesicle cycling and axon outgrowth
Source: Hum Mol Genet. 2013 Aug 15;23(1):90–103. doi: 10.1093/hmg/ddt401 (PMC3857945; doi:10.1093/hmg/ddt401)
Supplement: Supplementary Data [file supp_ddt401_ddt401supp.pdf]

Human Molecular Genetics
submitted

SUPPLEMENTARY MATERIALS

SYN2 is an Autism Predisposing Gene: Loss-of-function Mutations Alter Synaptic Vesicle Cycling and Axon Outgrowth

Anna Corradi[1§], Mauela Fadda[1,2§], Amélie Piton[3§], Lysanne Patry[3], Antonella Marte[1], Pia Rossi[1], Maxime Cadieux-Dion[3], Julie Gauthier[3], Line Lapointe[3], Laurent Mottron[3], Flavia Valtorta[4], Guy A. Rouleau[3], Anna Fassio[1,2], Fabio Benfenati[1,2§*], Patrick Cossette[3§]

[1]Department of Experimental Medicine, University of Genova, Viale Benedetto XV 3, 16132 Genova, Italy; [2]Department of Neuroscience and Brain Technologies, Fondazione Istituto Italiano di Tecnologia, Via Morego 30, 16163 Genova, Italy; [3]Centre of Excellence in Neuromics and CHUM Research Center, Université de Montréal, CHUM-Hôpital Notre-Dame, 1560 Sherbrooke Est, Montréal, Quebec H2L 4M1; [4]S. Raffaele Sci. Institute and Vita-Salute University, Via Olgettina 58, 20132 Milano, Italy

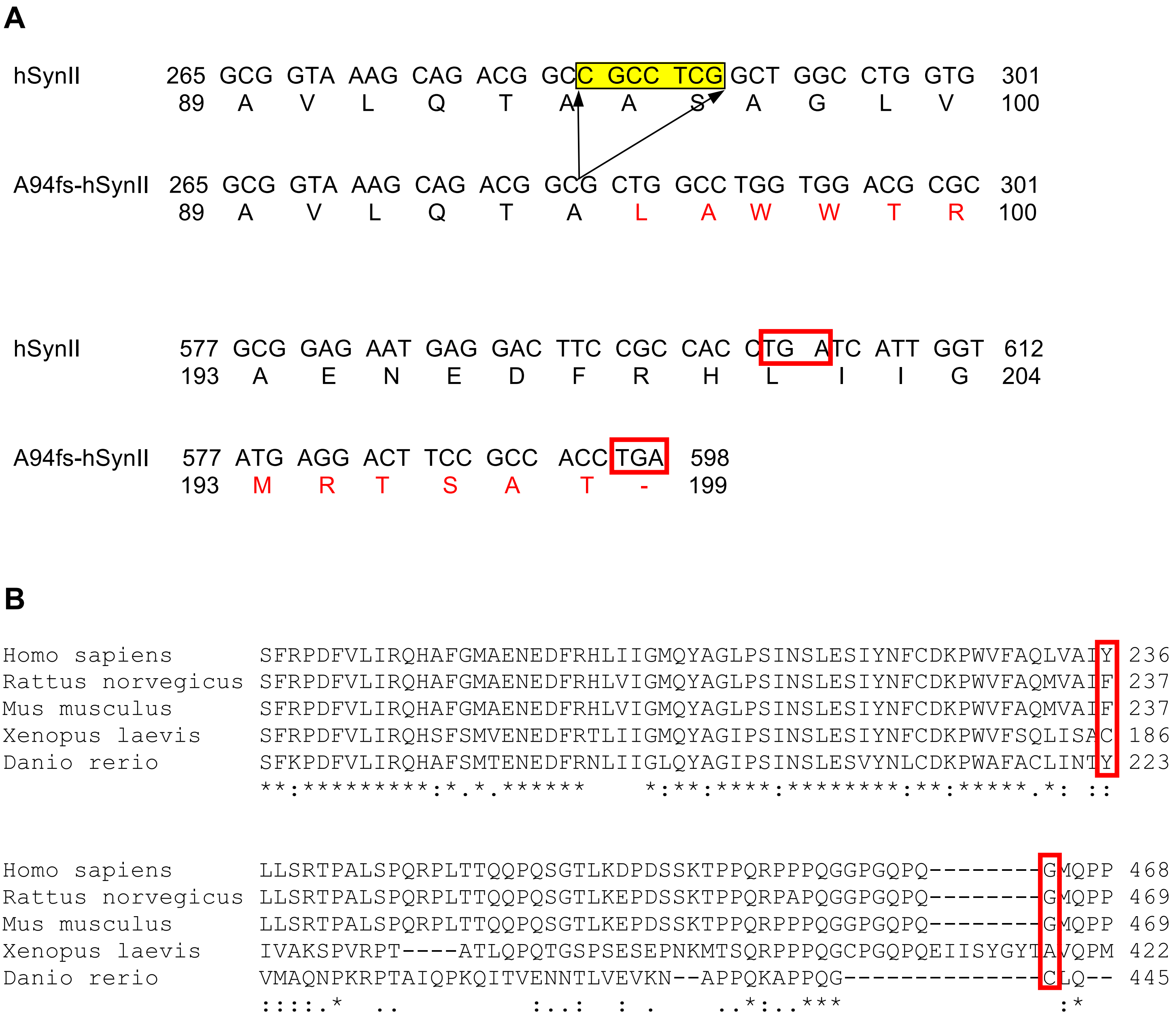

Supplementary Figure 1

Legend to Supplementary Figure 1.

A. The p.A94fs199X frameshift mutation is a CGCCTCG deletion in position 282-288 of the hSynII coding sequence (yellow box) that causes a precocious stop codon at position 598 (red box). The protein encoded by the mutated allele, if translated, would be truncated at position 199. The mutant protein shares with WT-hSynII only the first 94 amino acids, while the rest of the sequence before the truncation (95-198) is aberrant (red sequence). Nucleotide and amino acid sequences of hSynIIa, and the relative sequence in which the frameshift mutation occurs at position 94 leading to a truncated protein of 199 amino acids, are reported.

B. Degree of conservation of the hSynIIa mutated residues (Y236S and G464R) across vertebrate species. The multiple ClustalW2 aligment of SYNII proteins in various species shows total (*) or partial (: .) conservation of residues from *Danio rerio* to human. In mammalian species, G464 is fully conserved while Y236 can show a conservative substitution with F.

SUPPLEMENTARY INFORMATION

Clinical description of mutation carriers

R0024579 (Y236S)

This subject is a boy with unremarkable antenatal and perinatal history (including any stress or trauma during pregnancy) from a mixed ethnicity. Paternal grandfather and maternal grandmother are Polish and English of origin respectively. Maternal grandfather and maternal grandmother are French-Canadian and English respectively. The ages of the father and mother when the subject was born were 44 years and 48 years respectively. The mother and father had college and university education levels, respectively. The developmental history is as follows: smile at 2-3 months, sitting without support at 6-7 months, walking alone at 13-14 months, toilet trained for urine, but not stool, at 3 years of age, first words at 18 months and first phrase at 3-3.5 years of age. The subject has been tested negative for Fragile-X syndrome. He was referred for investigation at 4.5 years of age for the presenting concerns: poor speech, tantrums, spins, does not realize consequence of actions. He did not have lost skills (language, motor or social interest and responsiveness). There is no paternal family history of autism, learning problems or mental illness. The mother had 1 still birth at 23 weeks and 2 miscarriages. The mother's sister had a severe depression and obsessions. One maternal aunt and one cousin of the mother present psychiatric problems (in the spectrum of schizophrenia, but not diagnosed). The Autism Diagnostic Observation Schedule-Generic (ADOS-G) communication score is 4 (autism cutoff = 4, autism spectrum = 2), social interaction is 7 (autism cutoff = 7, autism spectrum = 4) and total is 11 (autism cutoff = 12, autism spectrum = 7). The Autism Screening Questionnaire (ASQ) score for this patient was 16 (score >15 = ASD). The overall impression and DSM-IV diagnosis of the subject is Autistic Spectrum Disorder, higher functioning.

R0024053 (p.A94fs199X)

This subject is a male of French-Canadian origin. Placental abruption occurred at 7 months of pregnancy. He was born by emergency caesarean section. At 6 months

of age he was hospitalized for asthma. He had two bilateral inguinal hernias, surgical cure during the first year. He sat at 7 months of age, walked at 11 months. He had amygdalectomy and adenoidectomy at 2 years of age. His behaviour was found to be abnormal with slowness and excessive quietness noticed at 7 months, as well as alarm signs for autistic behaviors around 1 year (e.g. look as if deaf, gaze avoidance, hand leading, visual perceptive interests, tantrum when adults seek contact, no facial expressions, avoidance of peer contact, sleep problems, self-injury). At 2.6 years of age the ADI was positive for autism (comm 14, thr 4, soc 28, thre 10, RIRB 9, thr 3), with a complete autistic picture including hand flapping and upright looking. The ADOS G was also positive for autism (comm 4 ,thr 4, soc 10, thr 7, Vineland: 19, 4 months). At 3 years of age, he had considerable improvement of social reciprocity, but still heavy speech delay. At 5.8 years of age, he exhibited speech impairment (phonological, oral and dyspraxia). He had delay in all aspects of language, including comprehension and syntax.

R0017205 (G464R)

This subject is a boy born in a refugee camp in Vietnam. He is diagnosed with autism. He had some traits of hebephrenic schizophrenia, but schizophrenia was later ruled-out. He has a mild thalassemia. The Autism Diagnostic Observation Schedule-Generic (ADOS-G) communication and social score is 20 (autism cutoff= 4-7, autism spectrum = 2-4). The Autism Diagnostic Interview (ADI) for social is 26; communication 14; stereotype 5 and developmental 4.
